# Expected challenges for memory clinics introducing disease modifying treatments in the Nordics

**DOI:** 10.1002/alz.088287

**Published:** 2025-01-09

**Authors:** Anders Gustavsson, Sebastian Palmqvist, Silke Kern, Jukka Saarinen, Vesna Jelic, Sohad F Ghadbhan, Björn Strindberg Lennhed, Jonas Alexander Jarholm, Alexander Rieem Dun, Alexandra Cooper, Mats Ekelund, Maria Ellgren, Tormod Fladby, Katarina Nägga

**Affiliations:** ^1^ Karolinska Institutet, Stockholm Sweden; ^2^ Quantify Research, Stockholm Sweden; ^3^ Memory Clinic, Skåne University Hospital, Malmö Sweden; ^4^ Clinical Memory Research Unit, Department of Clinical Sciences Malmö, Faculty of Medicine, Lund University, Lund Sweden; ^5^ Department of Psychiatry Cognition and Old Age Psychiatry, Sahlgrenska University Hospital, Mölndal, Göteborg Sweden; ^6^ Sahlgrenska Academy, University of Gothenburg, Gothenburg Sweden; ^7^ Neurology department, Vaasa Central hospital, Vaasa Finland; ^8^ Theme Inflammation and Aging, Karolinska University Hospital, Stockholm Sweden; ^9^ Dementia clinic of Funen, Neurological department, Odense University hospital, Odense Denmark; ^10^ The Memory Clinic, Falu hospital, Falun Sweden; ^11^ Akershus University Hospital, Nordbyhagen, Akershus Norway; ^12^ Quantify Research AB, Stockholm Sweden; ^13^ BioArctic AB, Stockholm Sweden; ^14^ Institute of Clinical Medicine, University of Oslo, Oslo, Oslo Norway; ^15^ Linköping University, Linköping Sweden

## Abstract

**Background:**

New disease modifying treatments (DMTs) offer a new treatment paradigm in mild cognitive impairment (MCI) or mild dementia due to Alzheimer’s disease (AD). This likely requires significant changes in patient management and need for added capacity for diagnosis, treatment and monitoring.

**Method:**

We surveyed eight selected memory clinics in the three largest and two smaller regions of Sweden, plus one in each of Denmark, Finland and Norway on their current practice, number of patients by diagnosis and available resources. Findings were compared to clinic assumptions on a future scenario where eligible patients with MCI or mild dementia due to AD are offered DMT.

**Result:**

Up to 3, but most often less than 1, incident patients in 1000 inhabitants per year are seen at surveyed memory clinics, of which on average about 40% have MCI or mild dementia due to AD. This constitutes not more than a few percent of the 10‐30 persons in 1000 inhabitants expected to have AD at this stage in the Nordics today. Most patients get amyloid biomarker confirmation as part of their diagnosis, while there are long wait times for radiology, in particular magnetic resonance imaging.

**Conclusion:**

Most patients expected to be eligible for DMTs in AD are not referred to memory clinics today, but are managed in primary care or are not presenting with cognitive complaints to health care at all. Therefore, the main challenge is likely not to treat patients already seen at memory clinics, but to manage a potential increase in patients that would benefit from DMTs. New referral patterns and health care seeking behaviors are therefore expected to determine the need for investments in additional capacity to diagnose and treat eligible patients.

